# Potentiation Physiologically Proven

**Published:** 1893-05

**Authors:** Gustav Jaeger

**Affiliations:** Stuttgart, Germany


### POTENTIATION PHYSIOLOGICALLY PROVEN.

PROF. DR. GUSTAV JAEGER, STUTTGART, GERMANY.

[Translated from the Leipzig *Allegm. Homœp. Zeitung,* Vol. 125, No. 1 and 2. by B. Fincke, M. D.]

Continued from page 373 (Sept., 1892).

(c.) *The lower potencies of the Alkali-salts.*

The reasons why I have measured the potencies below the point of indifference, first and separately from the higher ones are the following:

(1.) I wanted to set myself right as quickly as possible as to whether such a laborious and time-taking work as the measurement of more than one hundred substances were worth the trouble; for this purpose the measurement of the lower potencies was sufficient.

(2.) The toxicology and posology working for scholastic medicine have experiences on the lower potencies, because they have proved the poisonousness of these substances. The neural analysis of the lower potencies could be controlled by them. It also gives information about the relations of poisonousness if not about all, yet about one part of them—the *paralytic* one—and if now the result of the neural analysis coincides with the experiences of the toxicologists, this is *per se* already valuable. But then it throws a clear light upon the results of the measurement of the *higher* potencies, and in this way:

Neural analysis measures exactly like a thermometer, two values contrary to a point of zero. *Paralysis and animation* are opposed to each other as *cold* and *heat.* If an optician manufactures a thermometer he determines the point of zero and the boiling point by which he obtains the scale for the degrees of *heat,* he now immediately draws conclusions from it upon the degrees of *cold,* and if somebody would maintain that the thermometer could measure the degrees of heat, but not those of cold? This were sheer nonsense; he would surely be declared crazy.

Thus, also, it is with neural analysis, if it indicates the relations of poisonousness which express themselves in values of paralysis correctly—*i. e.,* in accordance with the general experience—then, also, the opposite numbers, the values of *animation* must be real, and cannot depend upon accident, imagination, or what else may be the phraseology of the gentlemen critics.

This is the main reason why I have first applied neural analysis to the lower half of the scale of the nerve-actions, and dedicated to them also the following tables:

### III Table. The Lower Potencies of the Kali-salts.

| POTENCY. | 3 | 4 | 5 | 6 | 7 | 8 | 9 | 10 | 11 | 12 | 13 | 14 | 15 | SUMS OF THE MINUS VALUES. |
|---|---|---|---|---|---|---|---|---|---|---|---|---|---|---|
| Ka.-jod. | — 2 | — 1 | — 3 | +11 | +17 | +28 | +24 | | | | | | | 6 |
| " carb. | —37 | —19 | —16 | —20 | — 8 | + 3 | +10 | | | | | | | 100 |
| " sulph. | —40 | —13 | —15 | —19 | — 8 | + 2 | + 8 | | | | | | | 95 |
| " chlor. | —40 | —23 | —13 | —14 | —10 | + 6 | + 9 | | | | | | | 100 |
| " nitr. | —43 | —22 | —14 | —14 | —12 | + 4 | +13 | | | | | | | 105 |
| " phosph. | —43 | —28 | —26 | —25 | —28 | —20 | —12 | —12 | —11 | — 8 | — 7 | — 2 | | 222 |
| " brom. | —70 | —45 | —33 | —31 | —26 | —32 | —26 | —25 | —28 | —19 | —17 | —13 | —7 | 372 |
| Mean. | —39 | —21 | —17 | —15 | —11 | — 1 | + 4 | | | | | | | 104 |

(1.) Formally the following is to be said on the foregoing table:

(*a*) The upper horizontal column gives the potency-numbers, which run from three to fifteen, once because I had examined in all these measurements the 1st and 2d potency, then because the table must reach so far till the most obstinate salt, the Kalium-bromatum, arrived at its point of indifference.

(*b*) Now in seven horizontal columns follow the numbers for the seven measured salts. How far in every horizontal column the *low* potencies reach can partly be seen from the sign of the numbers, which for the *low* potencies is the *minus* sign, for the *higher* potencies the *plus* sign, partly from the fact that a vertical line separates in every horizontal column the minus values from the series of the plus values. I call this the *line* of *indifference.* It lies in Kalium-jodatum between the numbers of the 5th and 6th potency, in the four next salts between the 7th and 8th, in Kali-phosphoricum, between the 14th and 15th ; in Kalium-bromatum, behind the 15th.

(*c*) The last horizontal column marked *mean* gives for every potency a mean number formed from seven different salts for the sake of comparing the groups of the Kali-salts with that of the Natrum-resp., Ammoniac-salts. The formation of this mean number, of course, rendered it necessary to note in the table also the plus value of a part of the higher potencies of those salts which previously reach the point of indifference, and this so far till the concerning mean number itself resulted in a plus value, which here happened at the 9th potency. Where the potency yielded throughout minus values they were added and divided by seven. Where the vertical column, as the case is after the 6th potency, contains values of either kind, the minus and plus values were added separately, the difference formed and divided by seven.

(*d*) Since with the toxical property of a substance the question is not only what paralytic effect it exerts upon a certain potency, but also how long its poisonousness opposes the method of attenuation—in other words, how high its point of indifference is, a *sum* had to be formed from the minus values—*i. e.*, the paralytic effects of all the lower potencies of every salt and for the series of the mean numbers in order to obtain a *single* number for *both* factors. These numbers are contained in the last vertical column.

(2.) On the contents of the table is to be said next that the course of the line of indifference divides these seven Kali-salts into three strikingly different groups.

The I group is formed by the Kalium-jodatum with quite an insignificant poisonousness which shows itself in the table in two ways: (1.) In the low paralytic numbers of the 3d, 4th, and 5th potency. (2.) In the circumstance that the change from paralysis into animation lies already between the 5th and 6th potency. Both together express themselves in that the sum of the minus values has only 6 points.

The II group consists of four substances, Kali-carbonicum, Sulphuricum, Chloricum, and Nitricum. Their third potencies have paralytic values of 37 to 43, and the point of indifference lies with all them between the 7th and 8th potency, therefore, higher by 2 potencies than with Kalium-jodatum, and the sum of the minus values lies with them between 95 and 105.

The III group is formed by Kali-phosphoricum and Kalium-bromatum, which, indeed, are so different that every one could form a group by itself. That which is common to both and makes them different from group I and II is the exceedingly high position of the point of indifference. Whilst in Kalium-jodatum it lies between the 5th and 6th potency, in group II, between the 7th and 8th, it is in Kali-phosphoricum, between the 14th and 15th potency, in Kalium-bromatum, between the 15th and 16th potency. In accordance with this, the sum of their minus values is also more than double as high as with group II. The difference within this 3d group consists in this: whilst they in regard to the position of the point of indifference coincide very nearly, they differ remarkably in the *gravity* of the paralytic effect. The difference in the 3d potency is 27, in the 4th potency 17. This great difference repeats itself again from the 8th to 14th potency, and the cause of it is the great difference of the sum of the paralytic values. Whilst Kali-phosphoricum concludes its series with 222 points, Kalium-bromatum reaches the number 372 as the most paralytic of all the Kali-salts.

If we compare the neural analytic result with the experiences which have been obtained in regard to these Kali-salts in another way, the following is the result:

(*a*) The contrast between Kalium-jodatum and Kalium-bromatum, established by neural analysis, corresponds exactly with the known facts. Kalium-bromatum is the main medicament of allopathy against epilepsy—*i. e.*, not as the remedy, but as the palliative—a property which it owes to the circumstance that it cancels the cramp irritation of the nerve system, which must be considered as a paralytic influence. Kalium-jodatum, on the contrary, belongs to those medicaments of allopathy to which it ascribes the most vigorous and far-reaching actual healing action—*i. e.*, the capability to induce the dissolution and absorption of morbid products and to which it also clung persistently in the times of the strongest nihilism. This accords very well with the circumstance that this salt oversteps the point of indifference already between the 5th and 6th potency, and after this potency acts *animating*, therefore, also *healing*, that, also, in giving allopathic doses if they are not repeated too often, an animating degree of attenuation can rapidly be reached by potentiation in the organism.

(*b*) The middle position of group II corresponds perfectly with the experience. Of its relatives no such paralytic actions are known as of Kalium-bromatum, and no such healing actions as of Kalium-jodatum.

(*c*) Furthermore, it corresponds with experience, that between the substances of group II no striking differences are known in regard to poisonousness ; hence, the fact of the table that their paralytic numbers and the position of the point of indifference are very near each other. The latter fact, the great correspondence of the four Kali-salts of group II, has induced me in the measurement of the higher (animating) potencies of the Kali-salts to abstain from the proving of all four members of this group, and to confine myself to the measurement of Kali-carbonicum as their representative.

(*d*) On the position of Kali-phosphoricum, as obtained by the neural-analytical measurement, the following is to be said : An accordance of neural analysis with other experience furnishes the fact that Kali-phosphoricum is not to be found in the list of the official medicaments, whilst all other six Kali-salts of our investigation enjoy this honor, yet not all for the same reason, viz. : Kalium-bromatum, on account of the high paralytic effect which it exerts on the nerves, and Kalium-jodatum, on account of its high dissolving, expelling power. As to the reason which caused the school-medicine to receive the Kali-salts group II among the medicines, only this can be said as the result of neural analysis : The paralytic numbers 95 to 105 give them a middle position, they are alluring neither as animating nor as paralytic medicaments, otherwise they are not so paralyzing that they could not be used on account of other properties and without evident danger of poisoning. We now come to the question: Why has the allopathic healing art not used Kali-phosphoricum for healing purposes? This is answered by the neural-analytical result, as follows: with the number 222 this salt stands in the midst between Kalium-bromatum (372) and group II (100); that is all. For if the allopathic physician wants to exert a paralytic influence he recurs to the medicament which corresponds mostly with this indication, and this is in this case Kalium-bromatum and not Kali-phosphoricum. On the other hand, if he desires to attain ends for which he applies salts of group II, he shall long for those with which his indication can be best fulfilled without disagreeable by-effects, and thus he shall abstain from the use of Kali-phosphoricum because its nervous by-effect is to easily produce paralytic phenomena. In this way the accordance of neural analysis with other facts is restored. Unfortunately, the circumstance, that Kali-phosphoricum is not officinal, is the cause that in the literature of the toxicologists, nothing can be found for our purposes.

### IV Table. The Lower Potencies of the Natrum-salts.

| POTENCY. | 3 | 4 | 5 | 6 | 7 | 8 | 9 | 10 | SUM OF THE MINUS VALUES. |
|---|---|---|---|---|---|---|---|---|---|
| Na.-mur. | — 5 | + 6 | + 7 | +23 | | | | | 5 |
| " nitr. | —10 | + 2 | +10 | +21 | | | | | 10 |
| " carb. | —14 | —16 | — 2 | + 5 | | | | | 32 |
| " sulph. | —19 | — 8 | — 4 | + 4 | | | | | 31 |
| " phosph. | —25 | —31 | —25 | —20 | —14 | —10 | + 1 | +7 | 125 |
| " brom. | —35 | —34 | —30 | —22 | —16 | —22 | —13 | —8 | 180 |
| Mean. | —18 | —12 | — 6 | — 2 | | | | | 38 |

Also here, as with the Kali-salts, three groups show themselves distinctly.

The I group is formed by kitchen-salt and saltpetre, both of which show only in the 3d potency paralytic numbers, and already in the 4th give animating effects which also far surpass Kalium-jodatum in lack of poisonousness, though the sum of their paralytic values show no considerable differences. With this in the most evident manner agrees the incontestable doctrine of experiences that to these two salts belongs the lowest degree of poisonousness, the fact that these two salts are the only alkali salts which we use as seasoning for our food and which we can use on account of their indifference or non-poisonousness. Also the difference between kitchen salt and saltpetre which allows the use of kitchen salt in greater quantities than of saltpetre without direct damage, appears clearly in the first numbers of the table; the 3d potency gives for kitchen salt 5%, for saltpetre 10% paralysis; the 4th potency for kitchen salt 6% animation, for saltpetre only 2%. This at the same time expresses that the point of indifference for kitchen salt of the 3d potency lies nearer than for saltpetre. When the solution of kitchen salt for injections and in microscopy considered as indifferent (physiologically) has been determined to be 0.6%, which, according to neural analysis, produces paralytic effects; this does not militate against its correctness; for it is a fact which will occupy us later extensively, since I can furnish proofs by numbers that these substances act more powerfully by inhalation than in any other way.

The II group is found by Natrum-carbonicum and sulphuricum. The experiential fact that these two salts are much less harmless than kitchen salt and saltpetre as to preclude their use as seasoning of food, agrees with the numbers of the table exactly. They show that the indifference with them appears first between the 5th and 6th potency, therefore two potencies higher than in group I, and when we look at the paralytic numbers of the food salts: kitchen salt 5, saltpetre 10; on the contrary, Natr-carbon. 32, Natr-sulph. 31, we have an expression in numbers as well for the difference between groups I

and II, as also for the great simility of Natr-sulph. and carbon which corresponds entirely with the known facts.

III group. This is exactly formed as with the Kali-salts, from the phosphoric and bromate-salt, and also again exactly with the difference as with the Kali-salts, viz., that the Brom.-salt is more paralytic, different and poisonous than the phosphorous-salt; with the latter the indifference lies between the 8th and 9th potency, therefore higher by three potencies than in group II, and with the Brom-natrium it occurs first between the 10th and 11th potency, is therefore higher by five potencies than in Group II. The sum of the paralytic numbers is with Natr-phos. 125, with Natr-brom. 180 and the exactitude of neural analysis is remarkable: the sum of the paralytic numbers is with Kal-phosph. 222, with Kali-brom. 372. Almost exactly the same proportion with Natrum the Phosphorus and Brom.-salts are as 12.5 : 18, with Kali as 11.1 : 18.6 and such a wonderfully working method is looked upon since almost ten years by the entire learned world with a shrug of the shoulders and hands in the pockets—but we rather not say it. Humboldt says: "In Germany *netto* two centuries are required to abolish a stupidity, one to find it out, another to set it aside." That the same is valid also for the introduction of inventions history proves in many instances.

*Comparison of Kali and Natrum-salts.*—For this purpose serve the mean numbers of the last horizontal column in Tables III and IV, especially the there given sum of the paralytic values, which with the Natrum-salts is 38, with the Kali-salts 104, two numbers which are about as 1 : 3—*i. e.*, if we reckon as above, the Kali-salts are three times as poisonous as the Natrum-salts. Now nobody will contend that all the facts known speak for the greater poisonousness of the Kali-salts, viz., of their ability to produce paralyses, especially paralysis of the heart. This has especially come to-day in the provings of the extracts of meat which are so rich in Kali-salts.

Dr. E. Kroener, homœopathic physician in Potsdam, was so kind as to examine the toxicological literature in regard to statements in this relation and sent me the following data:

(1) Natrium-salts are in the triple-fatal doses of the Kalium-salts still unpoisonous when injected in the veins.

(2) Of Chlor-natrium by the mouth the fatal doses for man is 250–500 gr.; for animals (probably rabbits) 10–15 gr. Of Chlor-kalium 1 gr. kills a rabbit instantaneously.

(3) Natrum-carbonicum relatively unpoisonous; of Kali-carbonicum two fatal cases by 15 gr. are stated.

(4) Natrum-sulphuricum relatively unpoisonous; Kali-sulphuricum one fatal case after 10–20 gr.

(5) On the two saltpetres there were only two statements. Natrum-saltpetre killed dogs in doses of 6–7 gr.; Kali-saltpetre men with 8 gr. Since the body of man is more voluminous than that of the dog, it also here appears that Natrum-salts, in order to force poisonous effects, must be taken in relatively larger doses than Kali-salts.

The foregoing culling from the toxicological literature of the school-medicine is indeed small, and the material in numbers—in comparison with mine—very poor, but yet sufficient to show that neural analysis and toxicological experience agree in that Kali-salts are by many degrees more poisonous than Natrum-salts.

Of the Ammoniac-salts, as will be seen, only four have been investigated, for the same reasons that, after furnishing the measurement of the lower potencies of the Kali-salts, I put aside three of seven and measured only the higher potencies.

These reasons are shortly, as follows:

(*a*) Regarding the Kali and Natrum-salts, I had convinced myself, that concerning the nervous effect they divide into three groups, of which, especially Group II, consists of such which in their action are so similar to each other that it is not worth while to measure them through all the potencies. To exemplify the method, I set aside three Kali-salts of this Group II, and with the Ammoniac-salts confined myself generally to a member of this group, viz., to the Carbonate. In order, however, not to reduce too much, I exempted the Natrum-salts, of which I originally had examined one less than of the Kali-salts, from the reduction.

### V. Table. The Lower Potencies of the Ammoniac-salts.

| POTENCY. | 3 | 4 | 5 | 6 | 7 | 8 | 9 | 10 | 11 | 12 | 13 | 14 | 15 | 16 | 17 | 18 | 19 | 20 | SUM OF THE MINUS VALUES. |
|---|---|---|---|---|---|---|---|---|---|---|---|---|---|---|---|---|---|---|---|
| Am-mur. | —34 | —22 | —25 | —30 | —23 | —19 | —10 | — 2 | + 4 | + 8 | + 9 | +10 | +12 | +15 | | | | | 165 |
| " carb. | —50 | —36 | —36 | —24 | —37 | —21 | —12 | — 7 | + 4 | + 3 | + 9 | + 9 | +10 | +15 | | | | | 223 |
| " phosph. | —64 | —44 | —46 | —41 | —26 | —47 | —32 | —31 | —20 | —15 | —11 | —12 | —13 | —17 | —13 | — 8 | | | 442 |
| " brom. | —63 | —43 | —45 | —40 | —35 | —38 | —32 | —29 | —29 | —25 | —18 | —23 | —22 | —33 | —24 | —21 | —15 | —8 | 543 |
| Mean. | —53 | —36 | —38 | —34 | —30 | —31 | —22 | —17 | —10 | — 7 | — 3 | — 4 | — 3 | — 5 | | | | | 293 |

(*b*) The Group III, consisting of the Phosphorous-salt and the Bromium-salt I did not want to reduce, because they are not so similar to one another as the salts of Group II, and thus the number 4 resulted.

If, in the above table, we consider only the position of the point of indifference, the Ammoniac-salts have not the division into three which is so sharply marked in the Kali and Natrum-salts. For both salts, which represent Groups I and II, have the point of indifference between the 10th and 11th potency.

If, on the contrary, we consider the numbers of the two salts, also here the difference of Group I and II distinctly appears, in so far as the Chlor-ammonium, with a paralytic value of 34 in the 3d potency, and with a sum of 160 is pretty less different than Ammon-carb., with paralytic value of 50 in the 3d potency and the sum of 223. This is the counterpart to the Natrum-salts where the combination with Chlorum likewise is decidedly less poisonous than the Carbonate. (See also the annexed table.)

The contents of the table are remarkable for the following:

(*a*) The correspondence with the Kali-salts, which consists in that the proportions between Group II and III are almost the same: as to *Kali* the point of indifference of Group II is between 7th and 8th, in Group III between 15th and 16th, therefore doubly as high; as to *Ammoniac* the concerning numbers are 9th and 10th in II, and 20th and 21st in III. The sums of the minus values are also almost equal. Kali-salt, Group II, 95–105; Phosphorus-salt, 222. Ammoniac-salt, Group II, 223; Phosphorus-salt, 442, therefore, both times a proportion of 1 : 2.

(*b*) The difference between Phosphorus and Bromium-salts repeats itself here almost in the same manner; at one time the point of indifference is higher in the latter, then the sum of the paralytic values is greater with the Bromium-salts, only this difference is proportionately smaller. If we examine the single numbers, we find that the paralytic values, up to the 10th potency, are almost equal with both, only with and after the 21st greater differences appear.

(*c*) The most remarkable fact of the table, that there are substances which even in the 18th potency, therefore in trillionth attenuation, yea even in the 20th potency, can act as *atmospheric poisons*, therefore in an attenuation in which chemistry proves itself perfectly powerless, can be considered only after we have finished the production of numbers.

### Comparison of the Ammoniac-salts with Kali and Natrum-salts.

A comparative view of the three tables teaches that of all the three groups the Ammoniac-salts have the greatest poisonousness, the greatest difference respectively hostility to life, and that in this they surpass the Kali-salts in almost all points.

(1) In the position of the point of indifference. With the *Natrum*-salts it moves between the 3d and 11th potency; with the *Kali*-salts between 5th and 6th; with the *Ammoniac*-salts between 10th and 21st.

(2) The mean numbers teach it in both ways: (*a*) in the numbers of the 3d potency, which, with the Natrum-salts, is 18, with the Kali-salts 38, with the Ammoniac-salts 48; (*b*) in the sum of the paralytic values: the sum of the six Natrum-salts is in the average 38; that of the Kali-salts, 104; that of the four Ammoniac-salts, 293.

The following statements in literature agree with the facts, that Ammoniac-salts are more poisonous, more different, than Kali-salts.

(1) For Chlor-kalium the dose prescribed is 1.0 to 2.0 gram.; for Chlor-ammonium, only 0.2 to 1.0 gram.

(2) For Brom-kalium the dosage commences with 0.5, for Brom-ammonium with 0.1, and at the same time it is stated that Brom-ammonium produces a stronger anæsthesis of the mucous membranes than Brom-kalium.

[TO BE CONTINUED.]

